# Multi-Criteria Decision Analysis (MCDA) Models in Health Technology Assessment of Orphan Drugs—a Systematic Literature Review. Next Steps in Methodology Development?

**DOI:** 10.3389/fpubh.2018.00287

**Published:** 2018-10-15

**Authors:** Aleksandra Baran-Kooiker, Marcin Czech, Coen Kooiker

**Affiliations:** ^1^Department of Pharmacoeconomics, Faculty of Pharmacy, Medical University of Warsaw, Warsaw, Poland; ^2^Department of Pharmacoeconomics, The Institute of Mother and Child, Warsaw, Poland; ^3^Warsaw University of Technology Business School, Warsaw, Poland; ^4^Independent Researcher, Warsaw, Poland

**Keywords:** MCDA, orphan drugs, rare diseases, EVIDEM, HTA

## Abstract

**Background:** Multi-criteria decision analysis (MCDA) is a decision-making tool that can take into account multidimensional factors and enables comparison of (medical) technologies by combining individual criteria into one overall appraisal. The MCDA approach has slowly gained traction within Health Technology Assessment (HTA) and its elements are gradually being incorporated into HTA across Europe. Several groups of scientists have proposed MCDA approaches targeted toward orphan drugs and rare diseases by including criteria specific to rare diseases. The goal of this article is to provide an overview of the current state of knowledge and latest developments in the field of MCDA in HTA for orphan drugs, to review existing models, their design characteristics, as well as to identify opportunities for further model improvement.

**Methods:** A systematic literature search was conducted in January 2018 using four databases: MEDLINE (Pubmed), EBSCO HOST, EMBASE, and Web of science to find publications related to use of MCDA in the rare disease field (keywords: MCDA/orphan drug/rare disease and synonyms). Identified MCDA models were analyzed, e.g., structure, criteria, scoring, and weighting methodology.

**Results:** Two hundred and eleven publications were identified, of which 29 were included after removal of duplicates. 9 authors developed own MCDA models, 7 of which based on literature reviews intended to identify the most important and relevant decision criteria in the model. In 13 publications (8 models) weights were assigned to criteria based on stakeholder input. The most commonly chosen criteria for creation of the MCDA models were: comparative effectiveness/efficacy, the need for intervention, and disease severity. Some models have overlapping criteria, especially in the treatment cost and effectiveness areas.

**Conclusions:** A range of MCDA models for HTA have been developed, each with a slightly different approach, focus, and complexity, including several that specifically target rare diseases and orphan drug appraisal. Models have slowly progressed over the years based on pilots, stakeholder input, sharing experiences and scientific publications. However, full consensus on model structure, criteria selection and weighting is still lacking. A simplification of the MCDA model approach may increase its acceptance. A multi-stakeholder discussion on fundamental design and implementation strategies for MCDA models would be beneficial to this end.

## Introduction

### Health technology assessment of orphan drugs

There is an ongoing debate if conventional health technology assessment (HTA) methods are still appropriate for orphan medicinal products (OMPs) and other highly specialized, innovative, expensive treatments (1–4). The one-size-fits-all approach of traditional cost-effectiveness analysis (expressed as cost per quality-adjusted life-year) no longer seems suitable for many new innovative interventions, leading to difficulties in the reimbursement process and delay in patient access to potentially valuable treatments. Two main characteristics of rare disease populations lead to structural methodological problems: 1. the rarity of these diseases which creates hurdles in setting up randomized clinical trials with enough statistical power to discern an overall treatment effect, and 2. disease heterogeneity, which causes problems with defining suitable endpoints and the generation of clinically relevant, quantifiable and reproducible treatment outcomes (5–7). The fact that the costs of these specialized interventions are often much higher than treatments for common diseases, makes difficulties during reimbursement assessment nearly inevitable. Drug manufacturers try to justify the high prices by the costly research and development path for these drugs and the small patient populations on which they try to generate a return of investment. Societies universally share the intention to effectively treat patients with a high medical need but at what cost? Healthcare budgets are limited, which is why HTA is used to assess the worth of various medical interventions and make coverage decisions.

Direct treatment costs and effectiveness are the main (and often only) criteria that are taken into account, as well as the impact on the healthcare budget (7). The effect of treatment interventions on the quality of life and indirect costs, such as patients' and/or caregivers' loss of productivity, are often not included (8–10). These factors are especially relevant for rare diseases, where effects beyond the direct treatment effect and indirect costs can be of relatively high importance (11) and this can lead to an unfair view of the actual cost-effectiveness of orphan drugs (ODs). Medical costs other than drug costs are often high for rare disorders, which usually start at an early age and require life-long special care, including many healthcare professional (HCP) visits, hospitalization and other treatments by specialists and caregivers (11, 12)^1^; (13). Although some factors that are relevant to rare diseases may be considered by HTA agencies, such as “unmet medical need” and “disease severity” (14), HTA bodies generally have their own set of rules, which are often not well defined. Some agencies have lower requirements for reimbursement of orphan drugs, e.g., France and Turkey do not require a full cost-effectiveness analysis and Germany uses lower evidence thresholds for OMP's in the AMNOG process. UK's NICE accepts higher incremental cost-effectiveness ratios (ICER) for orphan and ultra-orphan drugs (15– 17)^2^^,^^3^; (18)^4^. HTA and reimbursement decision-making processes frequently lack transparency and consistency, making it difficult for manufacturers, payers, patients, and society as a whole to discuss or justify reimbursement decisions or to compare HTA outcomes between countries and regions. MCDA could help in providing a structured, predictable, and transparent approach.

### Multi-criteria decision analysis (MCDA)

Multi-criteria decision analysis is a decision-making tool that can take into account multidimensional factors and enables comparison of medical technologies by combining individual criteria into one overall appraisal (19, 20). MCDA can facilitate complex decision-making processes and has been used in a range of industries since the 1960's e.g., in financial decision-making, geographical information systems, and environmental impact studies (21–23). Having realized the issues with the healthcare reimbursement decision-making process, several groups of scientists adapted the traditional MCDA model toward HTA. One such initiative is the EVIDEM framework (Evidence and Value: Impact on DEcision Making), created by Goetghebeur and Wagner et al. (24) in 2008, which aims to be an open-source toolkit for healthcare reimbursement decision-making and which is continuously updated and improved (10th edition in 2017)^5^. In 2016 a first adaptation of EVIDEM specifically tailored to orphan drugs was created, trying to capture effects beyond costs and treatment efficacy, such as impact on quality-of-life of both patient and caregivers, impact on indirect costs such as productivity loss, as well as societal criteria which are traditionally not considered by payers/health insurers (25). A similar initiative is the Transparent Value Framework (TVF) created by Hughes-Wilson, which was tested within the EU Mechanism of Coordinated Access to orphan medicinal products project (MoCA) in 2013 (26)^6^. The main recommendation of MoCA was to develop a coordinated mechanism between the 12 participating Member States and OMP developers to evaluate the value of OMPs. This process would be based on a so-called transparent value framework, to support the exchange of information enabling informed pricing and reimbursement decisions at Member State level. This can be considered a first attempt to implement multi-criteria analysis into reimbursement processes across the EU (26)^6^.

The MCDA approach has slowly gained traction with HTA agencies and elements are gradually being incorporated into HTA across Europe. In Hungary MCDA was introduced by a ministerial decree in 2010 for the evaluation of new hospital medical technologies (27). In the Italian region of Lombardy MCDA has been used since 2011 as a supportive HTA tool for diagnostic and medical devices, interventional procedures and medicinal products (incl. OMPs), called the VTS model (28). The current VTS model is based on EUNetHTA's *Core* HTA model (v3.0), with an incorporation of MCDA elements from the EVIDEM MCDA framework (v3.0) (29). This pioneering work of the Lombardy region is now being explored by other regions in Italy as well as at the national level (30).

MCDA is also starting to be utilized by researchers in the rare disease field, which has resulted in a list of scientific publications. *Palaska* published an overview of existing OMP MCDA models in 2014 (poster presentation) (31). The group of experts collaborating in the ORPH-VAL working group also listed some of the OMP MCDA models and created a set of principles which should be taken into consideration during OMP reimbursement processes (30). After conducting the literature review in January 2018 the authors came across a review article with a similar concept (32). However, the identified article included fewer studies and focused mostly on explaining the methodology of MCDA.

### The objective of the study

The goal of this article is to provide an overview of the current state of knowledge and latest developments in the field of MCDA in HTA for orphan drugs, to review the existing models, their design characteristics, as well as to identify opportunities for further model improvement. Given the speed at which the orphan drug HTA field is changing, the authors believe a publication with the latest information on the use of MCDA models in orphan drugs is warranted (e.g., 10 new studies concerning this topic were published in 2017). Focus was laid on studies that discuss the design and practical implementation of MCDA, e.g., the identification of relevant model criteria and weights, scoring methods, assessment, and comparison of drugs through MCDA appraisal.

## Materials and methods

A systematic literature search was conducted using four databases: MEDLINE (Pubmed), EBSCO HOST, EMBASE and Web of Science in January 2018. The search strategy was focused on the use of MCDA in HTA and reimbursement of orphan drugs and methodological aspects of the different models. As many reimbursement/HTA aspects can be expressed in various ways, a high-level search was conducted with only the keywords “MCDA,” “orphan drugs,” and “rare diseases” (and synonyms) to capture all possible relevant publications.

The following search strategy was applied in all four databases:

[MCDA or (“multi-criteria-decision-analysis”) or (“multicriteria decision analysis”) or (“multi-criteria decision analysis”) or (“multi criteria decision analysis”) or (“multiple criteria decision analysis”) or (“multiple-criteria decision analysis”) or (“multiple-criteria-decision-analysis”) or (“multicriteria analysis”) or (“multi-criteria analysis”) or (“multi-criteria-analysis”) or (“multiple criteria analysis”) or (“multiple-criteria analysis”) or (“multiple-criteria-analysis”) or (“multi-criteria decision making”) or (“multi-criteria-decision-making”) or (“multi criteria decision making”) or (“multicriteria decision making”) or (“multiple-criteria decision making”) or (“multiple-criteria-decision-making”) or (“multiple criteria decision making”)] AND [(orphan drug^*^) OR “OD” OR “OMP” OR “ODs” OR (OMPs) OR (orphan medicinal product^*^) OR (rare disease^*^) OR (rare disorder^*^)].

No time limits were imposed in order to spot trends over time (if any). Only full-text articles and abstracts published by 1st January 2018 written in English were included. All steps of the literature review (identification, screening, eligibility, inclusion, and data extraction) were performed by 2 independent researchers.

Included in this review were original research publications addressing the following subjects within the area of MCDA for OMP/rare diseases: model creation and adjustment, identification and definition of model criteria, weight elicitation, model validation through MCDA OMP appraisal/testing, and articles on discussing the impact of MCDA application on decision making.

Publications that did not address MCDA and OMPs in-depth or as the main subject, as well as publications on the subject of MCDA outside the field of HTA and/or reimbursement for OMPs (e.g., MCDA in benefit-risk assessments or used in other medical conditions than rare diseases) were excluded.

### Identification

The initial search resulted in 211 publications (from Pubmed *n* = 24; EBSCO HOST *n* = 98; EMBASE *n* = 55; web of science *n* = 34). No additional records were identified using other sources.

### Screening

One hundred and four records remained after removing duplicates. They were individually screened by title and abstract. Fifty seven records were excluded as they were not relevant to orphan drugs/rare diseases and MCDA in reimbursement/HTA field (e.g., concerning the use of MCDA in benefit-risk assessment or other medical conditions than rare diseases). Forty seven records were screened by reading full-text articles, out of which 13 were excluded which did not have MCDA as the main subject or in case the publication had a more theoretical nature (not research-based) or contained only limited information on MCDA.

### Eligibility

Thirty four publications were included in qualitative synthesis. The authors decided to remove an additional 5 abstracts which had overlapping results and outcomes with identified full-text articles.

### Inclusion

**Figure 1:**
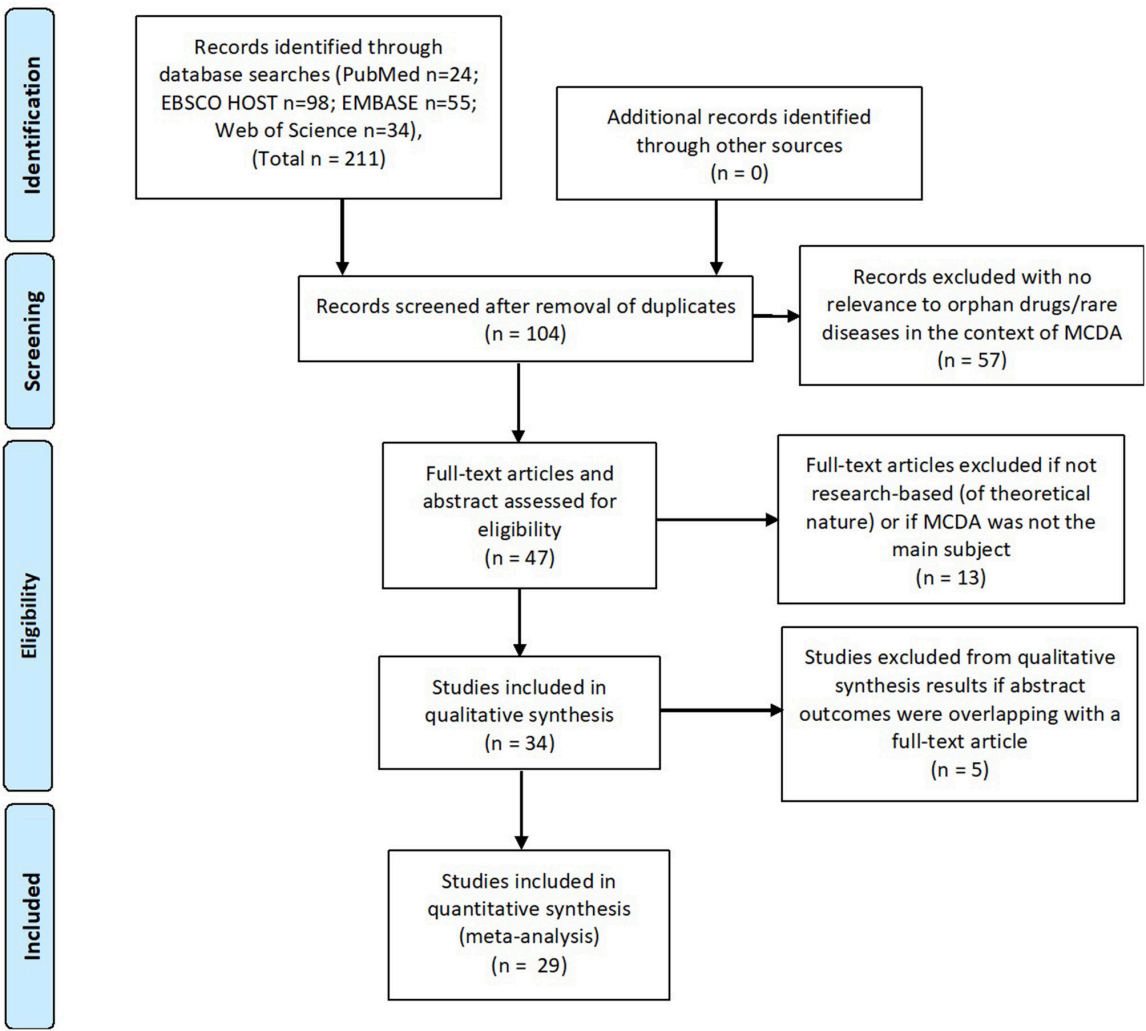
Systematic review flow (PRISMA).

Twenty nine publications were included in the systematic review, i.e., articles concerning MCDA in the field of reimbursement/HTA processes of rare diseases and orphan drugs: 13 full-text articles and 16 posters. Sixteen publications dealt with the creation and testing of MCDA models and 6 publications were of a more general nature describing MCDA principles (Figure 1). Data such as: title, author(s), year of publication, type of publication, goal of the article, methodology, involved stakeholders, methods used for criteria weighting, and scoring, a short description of results and information whether the model was tested/validated with a drug evaluation was extracted from the identified publications.

The systematic literature review was conducted and described in accordance with the PRISMA statement.

## Results

### Study characteristics

Of the 29 publications (8, 33–51), 18 were published in 2016 and onwards (the oldest being from 2011), which reflects the “novelty” of MCDA in OMP healthcare decision-making. All publications described the use of MCDA for assessment of OMPs in Europe, specifically in 10 European countries: Bulgaria, France, Germany, Italy, the Netherlands, Poland, Russia, Spain, Ukraine and the United Kingdom. Two publications discussed MCDA application in multiple European countries [Sussex et al. (37) and Wagner et al. (43)].

Sixteen publications focused mostly on defining the most appropriate model criteria [Wagner et al. (25) and Wagner et al. (43)], Hughes-Wilson et al. (33), Kolasa et al. (34), Iskrov et al. (35), Trip et al. (36), Sussex et al. (37), Schey et al. (42), Fedyaeva et al. (38), Paulden et al. (8), Palaska and Hutchings (31), and Piniazkho et al. (40)] but in 5 it did not lead to the creation of a defined MCDA model [Schlander et al. (52), Zhang et al. (53), Nemeth and Piniazhko (54), Korchagina et al. (55), and Hutching et al. (56)].

In 8 studies weights were assigned to criteria based on stakeholder input [Trip et al. (36), Sussex et al. (37), Gilabert-Perramon et al. (44), Garau et al. (45), Iskrov et al. (35), Fedyeva et al., Wagner et al. (43) Piniazkho et al. (40), and Piniazkho and Nemeth (41)] and in one publication weights were allocated by the authors [Wagner et al. (25)]. Two articles provided suggestions for MCDA score definition and calculation [Kolasa et al. (34) and Hughes-Wilson et al. (33)].

Trip et al. (36), Kolasa et al. (34), Schey et al. (42), Iskrov et al. (35), Paulden et al. (8), Sussex et al. (37), Wagner et al. (25), and Piniazkho et al. (40) developed their own MCDA models, primarily based on literature reviews that were intended to identify the most important and relevant decision criteria for the model. Hughes-Wilson (33), Fedyaeva et al. (38, 39), and Krysanova et al. (49) also built MCDA models, but information on the criteria definition and selection process is lacking. Wagner et al. (25) tailored the existing EVIDEM MCDA framework to orphan drugs, by adding sub-criteria specific for rare diseases into the model and by adjusting scoring scales.

Trip et al. (36), Iskrov et al. (35), Kolasa et al. (34), Sussex et al. (37), Schey et al. (46), Piniazkho et al. (40), Wagner et al (43), Schey et al. (46), Tony et al. (47), Badia et al. (50), Garau et al. (45), Jimenez et al. (48), Fedyaeva et al. and Krysanova et al. (49) performed a test of the MCDA model for orphan drug evaluation in their research. Hughes-Wilson et al. did not provide any information on testing the Transparent Value Framework MCDA model in the identified publication, however, the model was later tested within the EU MoCA project^6^. For more details refer to Table 1.

**Table 1:** The description of studies identified via literature review.

**Type of publication**	**Title**	**References**	**Goal of the article**	**Methodology**	**Stakeholders**	**Methods used for weighting criteria**	**Scoring methods**	**Results**	**Drug evaluation**
Full-text article	Can the EVIDEM Framework Tackle Issues Raised by Evaluating Treatments for Rare Diseases: Analysis of Issues and Policies, and Context-Specific Adaptation	Wagner et al. (25)	Analysis and further development of the EVIDEM MCDA framework to address rare disease issues and policies, while maintaining broader disease applicability.	Identification of specific issues and policies for RD's through literature review. Ethical and methodological perspectives and policies were integrated into the EVIDEM framework.	Authors of the article	Hierarchical Point Allocation (HPA). A theoretical example based on literature review was used but no research. Negative scoring introduced.	Intervention scored on each criterion using cardinal scoring scales, including zero scores in case of no value. Negative scores were included for comparative criteria to reflect worse outcomes or economic consequences than comparators. Impact of contextual criteria was measured qualitatively using a separate tool.	Analysis showed the framework integrates ethical dilemmas and issues inherent to appraising interventions for RD's but required further integration of specific aspects. Modification included addition of subcriteria to further differentiate disease severity, disease specific treatment outcomes, and economic consequences of interventions for RD's.	No
Full-text article	Drug Evaluation and Decision Making in Catalonia: Development and Validation of a Methodological Framework based on Multi-Criteria Decision Analysis (MCDA) for Orphan Drugs	Gilabert-Perramon et al. (44)	Adaptation and assessment of EVIDEM for OMP's in Catalonia.	Evaluation and decision-making procedures of CatSalut were compared with EVIDEM. EVIDEM was adapted to the Catalan context, focusing on OMP evaluation (PASFTAC program), during a PASFTAC Workshop. Criteria weighting was done with nonhierarchical and hierarchical methods. Reliability was assessed by re-test.	16 PASFTAC members (CatSalut).	5-point weighting & 5 points scale HPA	N/A	EVIDEM was found useful and feasible for OMP evaluation and decision-making in Catalonia. All criteria considered for the CatSalut Technical Reports and decision making were considered in the framework. The framework could improve reporting of some criteria (i.e., “unmet needs” or “nonmedical costs”). Some Contextual criteria were removed (i.e., “Mandate and scope of healthcare system”, “Environmental impact”) or adapted (“population priorities and access”) for CatSalut purposes. Independently of weighting techniques, the criteria viewed as most important for OMPs were: “disease severity”, “unmet needs” and “comparative effectiveness”, while the “population size” had the lowest relevance. Test–retest analysis showed weight consistency among techniques, supporting reliability over time.	No
Poster	Multi-Criteria Decision Analysis for Reimbursing Orphan Drugs: A Dutch Demonstration Study Using the Analytic Hierarchy Process Method	Trip et al. (36)	Demonstration if MCDA can support rational and explicit reimbursement decision making for OMP's in the Netherlands.	Analytic Hierarchy Process was used and Health Economics students were asked to weigh criteria used in drug reimbursement through a survey. Criteria were identified via literature review. Three different OMPs (alglucosidase alfa in infantile Pompe disease, canakinumab in cryopyrin-associated periodic syndromes and an investigational OMP) were assessed via these criteria. Criteria weights and scores were aggregated to an overall score. Rank-ordering on overall scores prioritized the reimbursement of the drugs. A separate feasibility of the AHP survey was done.	Health Economics students	Weighting by students (see Scoring Methods column)	Criteria weights and performance scores were aggregated into an overall score for each of the 3 orphan drugs. Rank-ordering on overall scores prioritized reimbursement.	9 criteria were identified and categorized in 4 domains; disease (burden of illness without treatment, life-threatening nature of the disease), drug (availability of other treatments, drug effectiveness, side effects and safety), financial aspects (annual drug costs per patient, budget impact, cost-effectiveness) and quality of evidence. The criterion ‘life-threatening nature of the disease' was given the most weight and budget impact the least. Alglucosidase alfa for infantile Pompe's ranked highest of the 3 OMP's examined, mainly due to its performance in disease and drug domains. The AHP survey was perceived as difficult by respondents, confirmed by poor consistency ratios.	Yes. Three different OMP's (alglucosidase alfa in infantile Pompe disease, canakinumab in cryopyrin-associated periodic syndromes and an investigational product in rare disease)
Poster	Assessing The Relationship Between Individual Attributes Identified In Review Of Multi-Criteria Decision Analysis (MCDA) Of Rare Diseases And Annual Treatment Costs In Rare Endocrine Disorders	Schey et al. (46)	Identification of MCDA criteria reported in literature and assess their impact on the total product “score” in relation to price.	A literature review was used to identify the most frequently cited MCDA attributes. From the leading attributes, the relationship between single attributes and the average annual treatment cost was plotted for several drugs for endocrine-related RD's. Annual treatment cost was based on UK prices for the average daily dose per patient.	N/A	No info	No info	The 3 most frequently mentioned attributes were “disease severity,” “treatment impact on condition,” and “level of research undertaken to support use of the product.” Disease severity was not shown to influence product price. Similarly, OMP's were not necessarily more expensive than non-orphan products. There was little discernible relationship between treatment “convenience” and average annual treatment cost. A trend was observed between market size and average annual treatment cost.	Rare Diseases And Annual Treatment Costs In Rare Endocrine Disorders
Full-text article	Multi-criteria Decision analysis for assessment and appraisal of Orphan Drugs	Iskrov et al. (35)	Creation of an MCDA model to assess OMP's by exploration of preferences for criteria weighting and scoring through a survey (143 stakeholders) and a focus group discussion in Bulgaria, and to test the model with 2 different medical technologies.	Model criteria were defined in a previous study among Bulgarian stakeholders. Weighting preferences for these criteria and performance scores were assessed through a stakeholder survey and a focus group discussion. A simple MCDA linear additive model was used, with an overall value combining weighted performance scores for all criteria (maxixum score: 100). Value assessment/appraisal was done from a societal perspective. The pilot model was tested with 2 hypothetical drugs and a reimbursement decision treshold was defined.	143 participants, incl. medical professionals, industry representatives, patients, health authority groups	100 points allocation	100 points allocation	Drug benefits/effectiveness were unanimously agreed upon as the most important group of reimbursement considerations. The study proved that strength of evidence might be a key criterion in orphan drug assessment and appraisal. Stakeholders consensually agreed on 70 or more points as a threshold for a positive recommendation and between 50 and 70 points for conditional reimbursement.	Yes, comparison of 2 OMP's
Full-text article	Multi-criteria decision analysis (MCDA): testing a proposed MCDA framework for orphan drugs	Schey et al. (46)	Exploration of the Hughes-Wilson MCDA framework for a range of OMP's, validated by the comparison of aggregate MCDA scores to pricing/average annual patient cost.	The Hughes-Wilson MCDA model was tested by using the suggested numerical scoring system on a scale of 1 to 3 for each criterion. Correlations between the average annual drug cost and aggregate MCDA score were tested and plotted graphically. Different weightings for each attribute were also tested. Further analysis was conducted to test the impact of including drug cost in the aggregate index scores.	N/A	3 scenarios for weights proposed by authors of the article	Numerical score from 1 to 3	Drug scores were plotted against the average annual cost per patient. For the drugs studied, the *R*^2^ was 0.7869 suggesting a strong correlation between the drug scores and the average annual cost per patient. The higher the drug's aggregate score, the more likely it was to have a high average per patient cost. Scenario analyses demonstrated that by applying different criteria weights, drug rankings changed, in particular for drugs with a higher average annual cost per patient.	Yes, Evaluation of 6 drugs for: Pulmonary arterial hypertension (PAH), Mucopolysaccharidosis VI (MPS VI), mucopolysaccharidosis II (MPS II), Paroxysmal nocturnal haemoglobinuria (PNH), Lennox-Gastaut syndrome (LGS) and Myelodysplastic syndromes (MDS).
Full-text article	Potential impact of the implementation of multiple-criteria decision analysis (MCDA) on the Polish pricing and reimbursement process of orphan drugs	Kolasa et al.(34)	Assessment of the impact of MCDA implementation on the Polish pricing and reimbursement process with regard to OMP's.	A 4-step approach was used, consisting of: 1. A literature review to select MCDA criteria and build an MCDA model. 2. A historical assessment of the HTA decision process and final outcomes for all 27 OMPs that had ever applied for reimbursement in Poland (21 positive, 6 negative recommendations). These drugs were then re-evaluated using the newly created MCDA and scoring methods. 3. Categorization of HTA recommendations and conducting an MCDA appraisal. 4. A comparison of HTA and MCDA outcomes. An MCDA outcome was considered positive if >50 % of the maximum number of points was reached (base case). In the sensitivity analysis, 25 % and 75 % thresholds were tested.	N/A	N/A	0–2 range	An MCDA model consisting of 10 criteria was created. 27 individual drug-indication pairs were reviewed and evaluated through and MCDA framework. Out of 27 cases related to 21 active substances, 6 received negative and 21 (78 %) positive recommendations for reimbursement by the Polish HTA Agency. Of the 27 cases, there were 12 disagreements between HTA and MCDA outcomes, the majority related to positive HTA guidance for negative MCDA outcomes. All drug-indication pairs with negative HTA recommendations were appraised positively in the MCDA framework. Economic details were available for 12 cases, 9 of which had positive MCDA outcomes. Among the 12 drug-indication pairs, 2 were negatively appraised in the HTA process, with positive MCDA guidance, and 2 were appraised in the opposite direction.	Yes, 27 drugs (21 reimbursed in PL and 6 with negative HTA recommendation)
Full-text article	A Pilot Study of Multicriteria Decision Analysis for Valuing Orphan Medicines	Sussex et al. (37)	An MCDA pilot to establish and apply a framework of weighted attributes to value OMPs	A literature review was performed on the natural history and burden of 40 RD's and of how payers assess treatment value, and 3 workshops were held with GlaxoSmithKline OMP managers, EU clinical and health economics experts, and RD patient representatives in the EU.	Clinical and health economics experts from France, Germany, Italy, Spain, and UK in April 2012; and representatives of rare diseases patient groups in the EU in August 2012.	Participants discussed in small groups how to allocate 100 weighting points across 8 attributes. Each group reached a consensus weight out of 100 for each criterion. Group weightings were reported to a plenary session and significant differences between groups were discussed, and each group could then revise its weightings.A consensus was reached in the plenary discussion for each attribute: all participants were content to accept an average of the groups' individual weightings, as amended following the plenary discussion, if there remained any difference in those weightings.	A 1 to 7 scale was chosen to permit sufficient discrimination without introducing an inappropriate impression of precision.	Eight non-monetary attributes were identified and weights agreed: 4 concerning the disease and unmet need and 4 on the treatment itself. Weighting was approximately evenly split between diseases attributes and treatment attributes. Patient group representatives gave greater weight than did the experts to patients' and carers' quality of daily life.	Yes,evaluation of 2 GSK drugs
poster	MCDA Approach To Ranking Rare Diseases In Russia: Preliminary Results	Fedyaeva et al. (38)	Creating and assessing the reliability and relative importance of 16 OMP related criteria (8 disease-related, 8 treatment-related) in an MCDA approach.	Refer to Goal of the article	85 experts were interviewed to estimate the importance of each criterion in the decision-making on financing MT for rare diseases. Respondents were 41 years on average (ranging from 23 to 64 years), and included 20 public servants, 16 health administrators, 32 practitioners, and 14 researchers. 44 respondents had a scientific degree.	10-point scale was used (10 points - major importance, 1 point minor importance). Mean estimates were calculated using descriptive statistics, then means were normalized.	N/A	The most important criteria were treatment characteristics - “Effect of treatment on quality of life” and “Effect of treatment on life expectancy” with 1 point each. The 2 least important criteria were disease characteristics - “Cognitive disorders as manifestations of the disease” and “Additional burden on the daily lives of care-givers” with 0.28 and 0.1 respectively. Treatment characteristics were more important for respondents than disease characteristics, therefore treatment characteristics should be given consideration when evaluating rare diseases to determine priority financing. The other criteria were not individually specified.	No
Full-text article	Appraising the holistic value of Lenvatinib for radio-iodine refractory differentiated thyroid cancer: A multi-country study applying pragmatic MCDA	Wagner et al. (43)	To assess the contribution of a range of criteria in the EVIDEM model to the value the OMP lenvatinib in radioiodine refractory differentiated thyroid cancer (RR-DTC) in country-specific contexts.	The study was designed based on analysis of the context in which lenvatinib will be appraised. Comparators were interventions indicated for the systemic treatment of RR-DTC (sorafenib). Since at the time of the assessment, reimbursement decisions for sorafenib had not yet been issued in target countries, watchful waiting was used as a second comparator. France, Italy and Spain were selected for country-specific assessments, as their HTAs involve multiple criteria. To collect insights from a broad range of perspectives and aim for a balanced appraisal, panels included a diversity of stakeholders. To explore the holistic value of lenvatinib, the EVIDEM framework (v2.4) was selected and all criteria were included. The study was designed to enable comprehensive appraisal (12 quantitative & 7 qualitative criteria).	Panels included policy decision makers, specialists, patient representatives, and methodologists with decision-making expertise, who were identified using predefined selection criteria and invited to participate in the study following local legal requirements.	Weight elicitation was performed using a 5-point direct weighting scale for the primary analysis and hierarchical point allocation (HPA) for sensitivity analyses. Weights were assigned to 19 criteria (12 quantitative, 7 qualitative) after interviews with relevant local stakeholders.	A constructed, cardinal scoring scale was used, ranging from 0 to 5 for non-comparative and from −5 to 5 for comparative criteria. Scoring was done based on a literature review, the sponsor's proprietary information and publicly available HTA data.	Comparative effectiveness, Quality of evidence (Spain and Italy) and Disease severity (France) received the greatest weights. Four criteria contributed most to the value of lenvatinib, reflecting its superior Comparative effectiveness (16–22% of value), the severity of RR-DTC (16–22%), significant unmet needs (14–21%) and robust evidence (14–20%). Contributions varied by comparator, country and individuals, highlighting the importance of context and consultation. Results were reproducible at the group level. Impacts of contextual criteria varied across countries reflecting different health systems and cultural backgrounds. The MCDA process promoted sharing stakeholders' knowledge on lenvatinib and insights on context.	Yes, drug comparison lenvatinib vs. sorafenib
Full-text article	Paying for the Orphan Drug System: break or bend? Is it time for a new evaluation system for payers in Europe to take account of new rare disease treatments?	Hughes-Wilson et al. (33)	Development of an assessment system based on multiple evaluation criteria for P&R of OMPs in the EU.	Design of an MCDA model with criteria defined by the authors.	N/A	N/A. No weighting performed. To be defined by individual governments.	Per model criterion 3 accepted price differentials were proposed (lower, medium, higher) based on the citerion score.	The authors propose the development of a new assessment system based on weighted evaluation criteria, which would serve as a tool for Member State governments, allowing different valuation of OMP's that fulfill all criteria vs. those with only some. An individual country could determine the (monetary) value that of each criterion, according to societal preferences, the national healthcare system and available resources. A new system could offer P&R decision-makers a tool to handle different OMP characteristics and redistribute national budgets in accordance with the outcome of a differentiated assessment.	No
Full-text article	Recommendations from the European Working Group for Value Assessment and Funding Processes in Rare Diseases (ORPH-VAL)	Annemans et al. (30)	Proposal of 9 principles to improve consistency of OMP P&R assessment in Europe and ensure that value assessment, pricing and funding processes reflect RDs and contribute to sustainability of healthcare and R&D.	A collaboration within European Working Group for Value Assessment and Funding Processes in Rare Diseases (ORPH-VAL) lead to defining 9 principles which should be used in HTA of OMPs.	European Working Group for Value Assessment and Funding Processes in Rare Diseases - ORPH-VAL (rare disease experts, patient representatives, academics, HTA practitioners, politicians and industry representatives).	N/A	N/A	The following Principles were established for value assessment and funding processes in RD's: (1) OMP assessment should consider all relevant elements of product value in an appropriate multi-dimensional framework, (2) P&R decisions should be founded on an OMP value assessment and reflect other considerations beyond product value (3) National P&R decision makers should take into account regulatory and HTA outcomes of OMPs undertaken in Europe (4) Assessment and appraisal of OMPs to inform national P&R decisions should incorporate RD expertise including both healthcare professional and patient perspectives (5) To accommodate uncertainty, value assessment and P&R decisions should be adaptive to new information (6) All eligible patients within the authorized label of an OMP should be considered in the national P&R decision, although different decisions on access may apply to different sub-populations (7) Funding should be provided at the national level to ensure patient access to OMPs (8) Evidence-based funding mechanisms should be developed to guarantee long-term sustainability (9) In the future there should be greater co-ordination of OMP value assessment processes at the European level.	No
Full-text article	Principles for consistent value assessment and sustainable funding of orphan drugs in Europe	Gutierrez et al. (51)	Proposal of principles to improve the consistency, effectiveness and sustainability of OMP value assessment in Europe, while maintaining flexibility and innovation, from the perspective of an OMP manufacturer.	N/A	N/A	N/A	N/A	10 principles were defined and grouped into 3 categories: Value Assessment, Innovation and Price and Sustainability of OMP Model. Value Assessment: (1): National P&R processes should acknowledge the EMA's assessment of therapeutic benefit (2): National Authorities should incorporate rare disease expertise within their local assessment processes (3): OMP assessment should consider all relevant elements of value (4): Value assessment methods for OMPs should incorporate multiple criteria (5): Value mechanisms should be flexible to accommodate evidential uncertainty at time of OMP approval Innovation and Price: (6): Adequate funding should be provided to ensure optimal patient access to OMPs and to incentivise research (7): OMP reimbursement decisions should be determined by benchmarking value and price against treatments with similar characteristics (8): If used, ICER thresholds should be modulated to reflect the specificities of rare diseases and OMPs Sustainability of OMP Model: (9): National authorities should develop adaptive and efficient processes to optimize use of real world data collected before and after value assessment 10: Rational and evidence-based funding mechanisms should be developed to guarantee long-term sustainability	No
Full-text article	Value-Based Reimbursement Decisions for Orphan Drugs: A Scoping Review and Decision Framework	(8)	Scoping and integrating social value arguments relating to OMP reimbursement within a coherent decision-making framework, to aid reimbursement decisions.	A scoping review of peer reviewed and gray literature was undertaken, consisting of seven phases: (1) identifying the research question; (2) searching for relevant studies; (3) selecting studies; (4) charting, extracting and tabulating data; (5) analyzing data; (6) consulting relevant experts; and (7) presenting results. The points within decision processes where the identified value arguments would be incorporated were then located. This mapping was used to construct a framework characterizing the distinct role of each value in informing decision making.	Patients, physicians, society	No info	N/A	The scoping review identified 19 candidate decision factors, most of which can be characterized as either value-bearing or “opportunity cost”-determining, as well as a number of value propositions and pertinent sources of preference information. Based on the results of the study the authors proposed a framework that separates value from cost bearing factors, includes stakeholder weighting (vs. comparators) for aiding coverage decisions for orphan therapies, centered around opportunity costs. The 19 candidate decision factors were: Disease prevalence (rarity), Disease severity, Identifiability of beneficiaries of treatment, Extent to which a disease is life-threatening or chronically debilitating, Evidence of efficacy/effectiveness, Magnitude of treatment benefit, Availability of alternatives, Treatment safety, Innovation profile, Societal impact of treatment, Impact of treatment on (equal) healthcare distribution, Socioeconomic policy objectives, Treatment cost, Budget Impact of treatment, Cost-Effectiveness, Feasibility of diagnosis, Feasibility of providing treatment, Industry/Commercial aspects, Legal considerations and Stakeholder preferences/value propositions.	No
Full-text article	Applying a Multi-criteria Decision Analysis (MCDA) Approach to Elicit Stakeholders' Preferences in Italy. The Case of Obinutuzumab for Rituximab-Refractory Indolent Non-Hodgkin Lymphoma (iNHL)	Garau et al. (45)	Using MCDA to obtain decision-criteria preferences of patients, clinicians and payers in Italy and to assess obinutuzumab for rituximab-refractory indolent non-Hodgkin lymphoma (iNHL).	An MCDA framework (EVIDEM V3.0) was used to elicit stakeholder preferences about the relative importance of decision criteria (weighting) and to assess the degree of achievement of obinutuzumab for rituximab-refractory iNHL in each criteria (scoring) via an online survey and structured meetings with each stakeholder group. The normalized weights and scores from each of the groups were combined with a linear function to calculate the intervention value score.	Clinicians, patients, and payers	Point allocation approach: allocate 100 points first across criterion domains and second across criteria within each clusters. Domain weights were combined with those within each domain and values normalized (to sum up to 1). Normalized weights and scores from each group were combined with a linear function to calculate the intervention value score.	Incremental criteria vs. comparator (related to health/non-health effects of the intervention, were measured on a scale from −5 to +5). Absolute criteria, related to disease characteristics (e.g., “disease severity”), were measured on a scale from 0 to 5.	Patients and clinicians expressed preference for interventions targeting severe conditions and ranked economic criteria among the 5 least important criteria. Payers expressed preference for treatments targeting populations with little or no effective treatment, which are less expensive than the comparator and with a high quality evidence. Obinutuzumab received high scores for “disease severity” and “type of therapeutic benefit” by all stakeholder groups. According to all stakeholder groups, the economic-related criteria (“comparative cost consequences – cost of intervention” and “comparative cost consequences – other medical costs”) for obinutuzumab obtained a negative score compared to its comparator bendamustine, whose patent recently expired.	Yes, obinutuzumab
Full-text article	Exploring Values of Health Technology Assessment Agencies using reflective Multicriteria and rare disease case	Goetghebeur et al. (57)	Tackling ethical dilemmas faced by reimbursement decision makers requires deeper understanding of values on which HTA agencies are founded and how trade-offs are made.	Representatives from eight HTA explored values on which institutions are founded using a narrative approach and reflective multicriteria (developed from EVIDEM, criteria derived from ethical imperatives of health care). Trade-offs between criteria and the impact of incorporating defined priorities (including for rare diseases) were explored through a quantitative values elicitation exercise.	Eight participants attended the workshop, each representing one HTA agency: Belgian Health Care Knowledge Centre (KCE); Canadian Agency for Drugs and Technologies in Health (CADTH); Health Technology Assessment Institute (IETS) Colombia; National Institute for Health and Care Excellence (NICE) UK; Lombardy Region Health Directorate, Italy; National Health Care Institute (ZIN) Netherlands; Norwegian Knowledge Centre for the Health Services (NOKC); and Instituto de Salud Carlos III, Spain.	N/A	N/A	Participants reported a diversity of substantive and procedural values with a common emphasis on scientific excellence, stakeholder involvement, independence, and transparency. Examining the ethical imperatives behind EVIDEM criteria was found to be useful to further explore substantive values. Most criteria were deemed to reflect institutions' values, with 70% of criteria reported by at least half of participants to be considered formally by their institutions. The quantitative values elicitation highlighted the difficulty to balance imperatives of “alleviating or preventing patient suffering,” “serving the whole population equitably,” “upholding healthcare system sustainability,” and “making decisions informed by evidence and context”, but they may help share ethical reasoning behind decisions. Incorporating “Priorities” (incl. for RD's) helped reveal trade-offs from other criteria and their underlying ethical imperatives.	No
Poster	Value assessment and pricing frameworks for rare disease treatments: new approaches from the literature	(31)	Summarizing RD specific value assessment and pricing frameworks proposed in the literature.	The literature review was conducted to identify papers that proposed specific frameworks for a) assessing the value of rare disease treatments or b) determining the price or reimbursement status of such drugs. Policy papers, commentaries and review articles were included. Clinical or economic studies of specific rare diseases and their treatments were excluded.	N/A	N/A	N/A	The literature review identified 1,034 papers including publications from conferences and an additional manual search. Of these, eleven papers were selected which included information on nine specific frameworks for assessing and/or pricing rare disease treatments. Criteria were categorized into 3 groups: disease characteristics, treatment characteristics, economic and healthcare system aspects. The most commonly used criteria were: disease severity, unmet need (lack of other alternatives), clinical efficacy, magnitude of clinical benefit, societal benefit from treatment, quality of evidence and safety profile of treatment. Four MCDA models included a budget impact or costs of the new treatment and three considered research and development effort as well as investment.	No
Poster	Methodological issues in MCDA for training needs: eliciting stakeholders' value preferences in Ukraine.	Piniazhko et al. (40)	Eliciting values and stakeholders' preferences in the decision making process on financing of cancer medication and orphan drugs in Ukraine.	A MCDA workshop was held with 24 national stakeholders, divided into 5 groups. Criteria were selected using “value trees” (Kanavos, Angelis, 2013) and weighting was performed. The sum of the criteria weights was set to 100.	24 Ukrainian experts involved in HTA and reimbursement	100 point weight allocation	N/A	Ranking of criteria, for cancer drugs: therapeutic effect 33 (SD ± 6.83), cost of treatment 23.6 (SD ± 13.59), burden of disease 17.4 (SD ± 9.0), safety 15 (SD ± 6.45), innovation level 11 (SD ± 4.47). For rare diseases: therapeutic effect 37 (SD ± 17.22), cost of treatment 30.8 (SD ± 20.26), safety 18.8 (SD ± 7.95), innovation level 9 (SD ± 3.42), burden of disease 4.4 (SD ± 3.03). Criteria for therapeutic benefits and costs had the highest values for stakeholders for both treatment options.	No
Poster	Practical issues of determining weights for criteria to be used in an MCDA Framework-Based On A Case-Study	Piniazhko and Nemeth (41)	Evaluating practical issues with determining the criteria weights of a MCDA framework and provide recommendations based on a case-study.	An MCDA workshop with 20 participants with healthcare-related backgrounds was held to assign weights to 5 criteria (Burden of disease, Therapeutic effect, Safety, Innovation, Costs), in 2 scenarios: 1 for oncology drugs and 1 for OMP's. The same participants were later assigned into 5 groups of 4 to discuss and agree on weights that represented the joint opinion of their group. The sets of criteria weights of individuals and groups from this workshop were assessed with descriptive statistical methods, e.g. standard deviation (SD) calculation.	20 Participants with various healthcare-related backgrounds	100 point allocation	N/A	Mean criteria weights of individuals and groups differed noticeably in some cases, e.g., 9.25 and 4.4 resp. for “Burden of disease” for cancer drugs. “Costs” had the highest SD values in both scenarios and both individual and group-based weighting. Contrary to expectations, the smallest criterion weight was not always lower for individuals than groups. Intra-group differences were significant, e.g., one group assigned the weight of 5 to “Therapeutic effect” while another set this weight to 60.	No
Poster	A common road map for rational clinical and policy decision-making: application of the MCDA based EVIDEM framework to growth hormone use in patients with Prader-Willi syndrome.	Tony et al. (47)	Application of a MCDA-based model to support and streamline policy and clinical decision-making in Canada for growth hormone (GH) therapy for Prader-Willi syndrome (PWS), a rare genetic disorder with serious long-term consequences including short stature and morbid obesity.	A literature review was used to identify and synthesize available evidence on GH for PWS for 19 criteria of EVIDEM. Evidence tables, quality assessment of studies, and synthesis of data by criterion were validated by experts using a web-based tool. The framework was used to develop CPG questions and structure development of international recommendations during a consensus workshop.	N/A	N/A	N/A	3 Levels of detail were generated for 13 scientific criteria of EVIDEM incl.: disease severity, size of population, therapeutic context and unmet needs, treatment outcomes (efficacy/effectiveness, safety, PRO's), type of treatment benefit at population and individual levels, and economic impact on medical and non-medical expenditures. Evidence for the 6 contextual and ethical criteria, incl. utility, efficiency, fairness, system capacity, stakeholder pressures, and political/historical context, was synthesized. CPG questions were developed following this format.	No
Poster	Development Of An Specific Evaluation Framework For Orphan Drugs Based On MCDA For Health Care Decision Making In Catalonia.	Badia et al. (50)	Developing an MCDA framework specific for Orphan Drugs in Catalonia, to facilitate and homogenize the assessment of OMP's by the Catalan Health Service (CatSalut).	A framework based on EVIDEM (v4.0) was developed for OMP evaluation and was tested and validated by representatives of the decision-making committee of CatSalut for 3 OMP's. The committee members rated individually the EVIDEM matrix for each drug assessment according to their preferences.	Decision-making committee of the Catalan Health Service (CatSalut	No info	No info	Several EVIDEM model criteria were removed or adapted accordingly after stakeholders' discussion. In the validation phase, some criteria were removed or not considered from standard EVIDEM (i.e., “size of population”, “non-medical costs”, “rarity” and “rule of rescue”) or adapted (“therapeutic benefit”) for CatSalut purposes. The assessment of 3 OMP's was conducted to rate the evidence matrix. Reflective discussion was seen as very relevant to support inputs for health decision-making processes on drug value and drug positioning. MCDA was considered useful methodology which adds transparency, predictability and allows a structured discussion and decision-making.	Yes, 3 OMPs: tolvaptan for autosomal dominant polycystic kidney disease, Alpha 1-antitrypsin for Alpha1-antitrypsin deficiency and eliglustat for Gaucher disease
Poster	Comparison of Methods to Assess the Relative Importance of Criteria in Multi-Criteria Decision Analysis: An Evaluation of Orphan Drugs in Russia	Fedyaeva et al. (39)	Estimating the relative importance of 10 identified MCDA criteria to evaluate OMPs, via 2 approaches: a direct rating weighting method (allocate points independently) and 'swing weighting (allocate a fixed total number of points over criteria). Direct weighting has limitations: performance range on criteria are not explicitly taken into account; weights are elicited independently, rather than by comparing importance. Swing weighting offers the potential to address these two concerns.	25 Healthcare decision makers in Russia were interviewed twice with a 7 day interval to estimate criteria importance. The direct rating method used a 10-point scale for each criterion independently. With the swing weighting method the most important criterion was allocated 100 points, and respondents were asked to allocate points to the other criteria to reflect relative importance of the ranges of performance. Descriptive statistics for each method were generated, and correlations of direct and swing weights were calculated.	25 Russian healthcare decision makers	Swing weighting: 100 point allocation and direct rating: 10 point scale	N/A	Ranking of criteria varied between the methods. When using direct rating, most importance was given to availability of treatment for a disease, and least importance to caregiver burden. When using swing weighting, respondents gave most importance to impact of treatment on life expectancy and least importance to the likelihood of occurrence of adverse events. Swing weighting resulted in greater differentiation between the criteria than direct weighting.	No
Poster	Determining the value of selexipag for the treatment of pulmonary arterial hypertension (PAH) in Spain by multi-criteria decision analysis (MCDA)	Jimenez et al. (48)	Ascertaining the value of the OMP selexipag in PAH compared to the main therapeutic alternative in Spain through MCDA.	Literature review (PICOT methodology, indexed, gray literature, primary and secondary search) completed with reference documents (regional and hospital evaluations, clinical guidelines). Real clinical practice experience with iloprost reported by clinicians given that data for selexipag (oral) and iloprost (inhalated) come from noncomparative (design, population and variables) clinical trials. MCDA EVIDEM (v4.0) framework was used in this study. The relative value contribution of selexipag vs. iloprost was obtained via criteria scoring and weighting by the panel.	45 Spanish national and regional eveluators incl. 32 multidisciplinary experts (cardiologists, pulmonologists, rheumatologists, internists, hospital pharmacists, decision-makers and patient representatives)	No info	scale −5 to +5	When compared with iloprost, selexipag was considered a new oral drug for PAH which adds value in the following MCDA quantitative criteria (scale −5 to +5): relative efficacy (2.3 ± 1.8), PRO's (2.5 ± 1.9), preventive benefit (2.8 ± 1.0), therapeutic benefit (3.0 ± 0.7), other medical costs (2.3 ± 1.6), other non-medical costs (2.1 ± 1.5). Based on clinical trial outcomes, selexipag was considered to have a potentially slightly worse safety profile (−0.3 ± 1.8) although AE's were considered transient, dose-dependent and easily managed with symptomatic treatment. MCDA allowed detailed analysis and discussion of overall value of selexipag in PAH treatment in a systematic, objective, pragmatic and transparent way relative to alternative treatment with iloprost in Spain. Reflective MCDA methodology favored discussion between panel members about what constitutes value in PAH which may be useful in drug evaluation and decision-making processes.	Yes, iloprost, and selexipag
poster	The multicriteria decision analysis of using tetrabenazine for patients with hungtington's disease in Russia	Krysanova et al. (49)	Performing MCDA to evaluate various aspects of the use of tetrabenazine (TBZ) for patients with Huntington's disease (HD).	Published clinical trials were analyzed to evaluate efficacy and safety of TBZ in HD. Value attributes (5 on the impact of rare disease and 5 on the impact of the drug) were identified from a literature review and expert's survey. Further experts assigned relative weights to the attributes in 2 groups. The rating scale ranged from 1 (least important) to 5 (most important). Experts rated TBZ against each attribute from 1 (worst score) to 7 (best score). In the end the weighted score for each attribute was identified.	Experts, details not specified	Scale 1–5	Scale 1–7	Experts considered the most important attributes to be: disease impact (scores 27.3 vs. 24.7). In both groups the most important attribute was evidence of treatment/clinical efficacy and patient clinical outcome (scores 6.67 and 6.00). The total weighted score for disease attributes was 85.78, and slightly lower for treatment attributes: 78.67.	Yes, tetrabenazine (TBZ)

### The EVIDEM framework

The EVIDEM framework was the most researched model in the identified publications: its applicability was assessed by: Jimenez et al. (48) in Spain (v 4.0), by Garau et al. (45) in Italy (v3.0), by Gilabert-Perramon et al. (44) in Catalonia (v3.0) and by Badia et al. (50), Tony et al. (47, 58) and Wagner et al. (43) in Italy, Spain and France (v2.4), respectively. In addition, Gilabert-Perramon et al. (44) compared the EVIDEM framework to the current HTA system in place in Catalonia (PASFTAC). The MCDA model created by Hughes-Wilson et al. (33) was tested by Schey et al. (46) and within the EU MoCA project.

Seven authors tried to identify criteria that should be taken into consideration during reimbursement and HTA processes of orphan drugs. Two of them [Gutierrez et al. (51), Annemans et al. (30)] emphasized the principles of MCDA development specific for orphan drug HTA in their articles. The rest were posters (52–56) without sufficient information to include them in this publication. Neither Gutierrez et al. (51) nor the authors of the *ORPH-VAL* group created a MCDA model, however, they selected and defined the main guiding principles for an OMP MCDA model that could be implemented in the EU. For the definition of the principles, Gutierrez et al. (51) took the perspective of a manufacturer of orphan drugs, whereas the ORPH-VAL group used a multi-stakeholder approach. Gutierrez et al. (51) defined a list of 10 principles that could help improve consistency, effectiveness and sustainability of orphan drug HTA models (no specific model was taken). Because the authors used the perspective of an OMP manufacturer, several principles were different from the other publications, e.g., the principle not to perform EMA's assessment of therapeutic benefit again during HTA on the national level. Goetghebeur et al. (57) conducted a questionnaire (based on EVIDEM) among health authority representatives from 8 countries to collect feedback on the mandates and values of HTA agencies, to examine ethical underpinnings of the HTA values and to explore trade-offs.

Detailed information on the identified studies can be found in Table 1 (57).

### Weighting

In 13 publications (using 8 different models) the authors assigned weights to the individual criteria in their models, based on stakeholder interviews via questionnaires or in workshops but also based on the authors' own insights. The most commonly used methods for weighting and scoring in the reviewed publications were: simple additive weighting (five-point scale) and hierarchical point allocation. Three criteria were consistently rated as the most important (i.e., having highest weight scores): disease severity/burden (Iskrov et al. (35), Wagner et al. (43), Gilabert-Perramon et al. (44), and Sussex et al. (37)], effectiveness/comparative effectiveness/therapeutic effect [Iskrov et al. (35), Wagner et al. (24, 25, 43), Gilabert-Perramon et al. (44), and Sussex et al. (37)] and “unmet medical need” (i.e., lack of alternative treatments) [Gilabert-Perramon et al. (44) and Sussex et al. (37)]. Other criteria that were rated high were quality/strength of evidence (of efficacy) (Gilabert-Perramon et al. (44), Wagner et al. (24, 25, 43), and Iskrov et al. (35)] and the safety/tolerability of the treatment [Gilabert-Perramon et al. (44) and Wagner et al. (43)].

### The comparison of criteria used in MCDA models

The most commonly chosen criteria for the creation of the MCDA models were:

- **Comparative effectiveness/efficacy** [8 models, 13 publications: Gilabert-Perramon et al. (44) and Wagner et al. (25, 43), Garau et al. (45), Iskrov et al. (35), Schey et al. (46), Kolasa et al. (34), Sussex et al. (37), Hughes-Wilson et al. (33), Trip et al. (36), Annemans et al. (30), Piniazkho et al. (40), and Piniazkho and Nemeth (41)] while a standard **cost-effectiveness analysis** was used in 3 (Trip et al. (36), Iskrov et al. (35), and Kolasa et al. (34)].
- **Unmet need/availability of therapeutic alternatives** [7 models, 11 publications: Gilabert-Perramon et al. (44), Wagner et al. (25, 43), Garau et al. (45), Iskrov et al. (35), Schey et al. (46), Kolasa et al. (34), Sussex et al. (37), Hughes-Wilson et al. (33), Trip et al. (36), and Annemans et al. (30)].
- **Disease severity** [7 models, 11 publications: Gilabert-Perramon et al. (44), Wagner et al.(25, 43), Garau et al. (45), Trip et al. (36), Iskrov et al. (35), Schey et al. (46), Kolasa et al. (34), Sussex et al. (37), Hughes-Wilson et al. (33), and Annemans et al. (30)].
- **Comparative safety/tolerability** [7 models, 11 publications: Gilabert-Perramon et al. (44), Wagner et al. (25, 43), Garau et al. (45), Trip et al. (36), Iskrov et al. (35), Kolasa et al. (34), Sussex et al. (37), Annemans at al. (30), Piniazkho et al. (40), and Piniazkho and Nemeth (41)].
- **Size of affected population** [3 models, 8 publications: Gilabert-Perramon et al. (44), Wagner et al. (25, 43), Garau et al. (45), Schey et al. (46), Kolasa et al. (34), Hughes-Wilson et al. (33), and Annemans et al. (30).
- **Quality of evidence** [6 models, 9 publications: Gilabert-Perramon et al. (44), Wagner et al. (25, 43), Garau et al. (45), Trip et al. (36), Iskrov et al. (35), Kolasa et al. (34), Sussex et al. (37), and Annemans et al. (30)].

Just as in *Gutierrez'* publications, several articles considered criteria that are relevant for OMP manufacturers: 3 publications (2 models) included the complexity of the manufacturing process [Schey et al. (46), Hughes-Wilson et al. (33), and Kolasa et al. (34)] and in 6 (4 models) the “level of research undertaken/innovativeness” was defined as a criterion [Schey et al. (46), Kolasa et al. (34), Sussex et al. (37), Hughes-Wilson et al. (33), Piniazkho et al. (40), and Piniazkho and Nemeth (41)].

A detailed list of all criteria included into the respective MCDA models is presented in Table 2.

**Table 2:** A list of criteria used in MCDA models. The EVIDEM model structure was used to compare criteria between models.

**List of criteria**	**EVIDEM framework model [Gilabert-Perramon et al. (44), Garau et al.(45), Wagner et al. (24, 25, 43), Badia et al. (50), Tony et al. (47), and Jimenez et al. (48)]**	**Monika Wagner et al (2015)**	**Trip et al. (36)**	**Iskrov et al. (35)**	**Hughes-Wilson model; [Hughes-Wilson et al. (33), Schey et al. (46)]**	**Kolasa et al. (34)**	**Sussex et al. (37)**	**Fedyaeva et al. (38, 39), Schey et al. (42, 46)**	**Annemans et al. ORPH-VAL group**	**Gutierrez et al. (51)**	**Piniazkho et al. (40)**
**Disease burden**	Disease burden	EVIDEM+ Contextual criteria + Sub-criteria (Impact on life-expectancy, Impact on morbidity, Impact on patient QoL, Impact on caregiver QoL, Other medical costs to healthcare system, Medical costs to patient, Patient/caregiver productivity, Costs to wider social care system, Non-medical costs to patients	Burden of illness without treatment	Disease burden				No full list of criteria	Patient economic burden, Healthcare system resources and budget, Healthcare system organization, Family/carer health-related QoL, Family/carer economic burden, Societal economic burden	No list of criteria but general principles (1): National pricing and reimbursement processes should acknowledge the EMA's assessment of therapeutic benefit (2): National Authorities should incorporate rare disease expertise within their local assessment processes (3): OMP assessment should consider all relevant elements of value (4): Value assessment methods for OMPs should incorporate multiple criteria (5): Value mechanisms should be flexible to accommodate evidential uncertainty at time of OMP approval (6): Adequate funding should be provided to ensure optimal patient access to OMPs and to incentivise research (7): OMP reimbursement decisions should be determined by benchmarking value and price against treatments with similar characteristics (8): If used, ICER thresholds should be modulated to reflect the specificities of rare diseases and OMPs (9): National authorities should develop adaptive and efficient processes to optimize use of real world data collected before and after value assessment (10): Rational and evidence-based funding mechanisms should be developed to guarantee long-term sustainability	Burden of disease
**Unmet needs**	Unmet needs		Availability of other treatments	Alternative	Available treatment alternatives/Unmet medical need/Use in unique indication or not	Therapeutics alternative (unmet medical need) and indication uniqueness	Availability of effective treatment options		Existing treatment options		
**Disease severity**	Disease severity		Life-threating nature of the disease	Disease severity	Disease severity/Level of impact on condition/disease modification	Disease severity	Disease survival prognosis with current standard of care and disease morbidity and patient clinical disability with current standard of care and Social impact of disease on patients' and carers' daily lives with current standard of care and		Survival/Life expectancy and Morbidity, Patient experience and health related QoL (related to disease characteristics)		
**Size of affected population**	Size of affected population				Rarity	Disease rarity			Rarity		
**Comparative effectiveness**	Comparative effectiveness		Effectiveness of drug	Clinical effectiveness	Level of uncertainty of effectiveness	Scientific evidence for clinical efficiency (level of uncertainty)	Evidence of treatment clinical efficacy and patient clinical outcome		Survival/Life expectancy and Morbidity, Family/carer health related QoL (related to effectiveness of treatment)		Therapeutic effect
**Comparative safety/tolerability**	Comparative safety/tolerability		Side effects and safety of the drug	Safety		Benefit from use of medicine (safety and adverse effects)	Treatment safety		Side effects		Safety
**Comparative patient-perceived health/patient-reported outcomes**	Comparative patient-perceived health/patient-reported outcomes								Patient experience and health related QoL		
**Type of preventive benefit**	Type of preventive benefit										
**Type of therapeutic benefit**	Type of therapeutic benefit			Health benefits					Treatment convenience		
**Costs**			Cost-effectiveness and Budget Impact	Cost-effectiveness and Budget Impact	Follow up measures (additional benefits and associated costs)	Cost-effectiveness and Budget Impact			Budget impact		Cost of treatment
**Comparative cost consequences - cost of intervention**	Comparative cost consequences - cost of intervention		Annual costs of the drug per patient								
**Comparative cost consequences - other medical costs**	Comparative cost consequences - other medical costs				Characteristics without direct cost impact				Patient economic burden		
**Comparative cost consequences - non-medical costs**	Comparative cost consequences - non-medical costs								Healthcare system resources and budget and Healthcare system organization and Societal economic burden		
**Knowledge about intervention**	Knowledge about intervention										
**Quality of evidence**	Quality of evidence		Quality of evidence	Strength of evidence		Scientific evidence for clinical efficiency (level of uncertainty)	Evidence of treatment clinical efficacy and patient clinical outcome		Quality of evidence and Uncertainty around value parameters		
**Expert consensus/clinical practice guidelines**	Expert consensus/clinical practice guidelines										
**Population priorities (Rare diseases vs Other priorities)**	Population priorities (Rare diseases vs Other priorities)			Vulnerable groups					Societal preferences		
	**Contextual Normative criteria:** Mandate and scope of healthcare system, population priorities and access, Common goal and specific interests, Environmental impact. **Feasibility contextual criteria:** Opportunity costs and affordibility, System capacity and appropriate use of intervention, Political/historical/cultural context										
**Innovativeness**					Level of research undertaken		Treatment innovation: scientific advance + contribution to patient outcome		Sustainability of innovation in rare disease		Innovation level
**Manufacturing complexity**					Manufacturing complexity	Manufacturing complexity					
**Other**				Life saving			Social Impact of treatment on patients' and carers' daily lives				

## Discussion

### Limitations of the study

One of the limitations of this review is its focus on publications specifically related to MCDA for orphan drugs, as this could have led to omission of publications with relevant and valuable content for this topic. A full review of MCDA in broader healthcare decision-making processes (e.g., for drugs for common diseases, medical devices or in hospital purchasing processes), in medical benefit-risk assessments or even the use of MCDA in other industries could be beneficial to get a complete picture of the methodology, its advantages and drawbacks. This could also give valuable insights on practical hurdles that exist for implementation of MCDA into decision-making processes, as well as relevant experiences and best practices.

The literature search strategy was designed to be broad with only 3 keywords, albeit designed to capture all possible variants of these keywords. A potential methodological shortcoming is that not all relevant publications may have been found due to articles missing the specific keyword of “rare disease” and/or “orphan drug,” e.g., research articles dealing with MCDA in a specific rare disease.

A systematic review format was used, no qualitative assessment was done of the different MCDA models, i.e., using a rating scale. Studies differ in design and scope and many models have both strengths and weaknesses. A valid comparison would require well-defined and objective metrics and valuation. Instead, the main focus has been laid on an assessment of the different criteria used in the MCDA models, general model structures, and possibilities for optimization of an MCDA model that is suitable, predictable and straightforward to use in practice. A more detailed analysis of model criteria and design considerations is given below.

### Commentary on the most frequently used criteria

#### Evaluation of model criteria—general commentary

Recently, a number of authors have expressed critical views on the current MCDA frameworks and pointed out several flaws and drawbacks of the used design and scoring methodology, as well as proposing new models or alterations to correct these faults (8, 59, 60). These critiques and the fact that new MCDA models or improvements to existing ones are continuously being developed, shows that the MCDA approach in healthcare has not yet reached maturity or general consensus. This could (partly) explain the slow or partial uptake by national HTA bodies in the EU. It is expected that any advancement in orphan drug MCDA will follow improvement and implementation of MCDA methodology in the broader healthcare setting. Incorporating orphan drug and rare disease-related factors into the current MCDA developments and discussions would be beneficial to prevent unnecessary model corrections for orphan drugs later on.

#### Simplicity and overlapping criteria

While it may seem methodologically or ethically desirable to include as many rare-disease specific components as possible into the MCDA model for orphan drugs, careful attention must be paid to criteria selection and weighting, to ensure meaningfulness, appropriate representation and to prevent overweighting and double-counting. This is also underlined by the ISPOR MCDA guideline, which specifies that criteria should be complete, non-redundant, non-overlapping and independent of preferences, as well as being unambiguous, comprehensive, direct, operational, and understandable (59).

Some identified MCDA models include the aspect of treatment effectiveness or cost-related aspects expressed in various manners, e.g., Iskrov et al. (35) uses the criteria “clinical effectiveness,” “health-benefits,” and “cost-effectiveness” (which includes an “effectiveness” component), which can amplify the outcome into a specific direction when items related to effectiveness are counted twice or more. *Trip*'s model contains drug effectiveness, cost-effectiveness and budget impact, as well as annual costs of the drug per patient, with overlap and therefore possible overweighting of costs and/or effectiveness in the scoring. Similarly, Kolasa et al. (34) took into account in their MCDA model the level of scientific evidence for “clinical efficiency” (level of uncertainty) and cost-effectiveness and budget impact, with clear overlap.

#### Criterion—benefits (EVIDEM, annemans)

Benefits is a broad “bucket” criterion that can contain a range of clinical advantages such as treatment convenience (as used in *Annemans'* model) and other contributions to patient care that can be relevant for disease management. The Benefits criterion can capture effects brought on by enhanced pharmaceutical formulations (e.g., improved patient-friendliness), less burdensome dosing schedules or other impact on treatment burden (e.g., fewer hospital visits needed), which can be relevant for certain rare disease treatments. The authors recommend a structured approach to the Benefits criterion with pre-specified sub-criteria. Including a field that captures “treatment compliance” would be beneficial, as this is an important factor for treatment success, and especially for costly orphan drugs the financial impact of non-compliance could be large. None of the identified models included compliance/adherence as a criterion.

Wagner et al's. (24, 25, 43) EVIDEM model contains the “Type of benefit” domain, which is used to quantify the “impact” of the preventive/therapeutic benefit for patients, e.g., a cure versus symptom relief. As discussed above, it can be argued that this field overlaps with the EVIDEM “Outcomes of intervention” domain that includes clinical effectiveness and safety. A similar situation exists for Patient-perceived health/PROs” (*EVIDEM, Annemans*) as this is a patient-reported measure of effectiveness that is additive and possibly overlapping. Models including PROs as a separate criterion should aim to prevent overweighting of clinical efficacy and other treatment benefits through PRO scores.

#### Criterion—disease population size (EVIDEM, hughes-wilson, kolasa, annemans)

While it is understood that rare diseases should be treated fairly and need a special approach, it is difficult to directly translate (a small) “population size” into a factor that is relevant to the value of a treatment and thus the MCDA outcome. Should the largest affected population receive priority treatment, which is a utilitarian view, or does the rarity of a disease justify a “compassionate” application of healthcare and therefore a higher priority above common diseases? This immediately complicates scoring and weighting: is less more or is more more?

The disease prevalence threshold in the EU for an orphan drug designation is well-defined at ≤ 5 per 10.000. Since it is expected that OMPs will most likely be compared directly with other similar (orphan) drugs, the relevance of population size on the MCDA outcome is limited. E.g. does a prevalence of 3 per 10.000 make a difference for the MCDA score over 2 per 10.000, even though it represents a 50% population size increase? Gilabert-Perramon et al. (44) also found that size of the population had the lowest relevance for decision-making (37).

Additionally, if the total budget impact of the treatment is included as a cost component in MCDA, it could be argued that the impact of “rarity” is amplified, e.g., possibly receiving a high score on “rarity” and a high score on “budget impact” (low budget impact, depending on a price of the product). While this can be the aim of a model, it should be considered during design/weighting and clearly expressed to assessors during scoring.

Since population size as such is difficult to score and might not have a relevant impact on the MCDA outcome anyway (especially when comparing orphan drugs), one can argue to not include this criterion, as Iskrov et al. (35), Sussex et al. (37), and Trip et al. (36) have done in their models. However, the authors consider that “Disease population size” could be a valuable factor in the model for certain stakeholders, as it could represent a direct incentive for manufacturers to develop drugs and other treatments for otherwise non-economical disease populations, which would otherwise be lacking.

#### Criterion—population priorities (EVIDEM, iskrov and annemans)

The argumentation for whether rare diseases should be treated differently by the population as a whole is essentially the same as described for “Disease Population Size.” It is difficult to generate an objective, meaningful value-score out of population priorities, and it should be considered that “Unmet need” or “Disease Severity” could also cover the “Population priority” aspect.

#### Criterion—unmet need (EVIDEM, trip, iskrov, hughes-wilson, kolasa, sussex, fedyaeva, annemans)

“Unmet (medical) need” is expressed differently in the various models, such as Availability of treatment (alternatives)/Unmet medical need or “Uniqueness of indication”, e.g., no strict definition exists. Unmet need can incorporate several defining criteria: patient population size, disease severity and lack of effective and/or approved treatments, which should be clearly defined, again to prevent overlap. Orphan drugs, by definition, target a disease population that has no effective and approved treatment; this is a requirement from regulatory agencies as EMA and FDA to be granted an orphan designation (exception: when a drug shows “significant benefit” over an existing orphan drug). In some cases, food products or nutritional supplements can be used to manage or alleviate symptoms of the disease (e.g., genistein for Sanfilippo syndrome) or food for special medical purposes (e.g., elimination diets) and OTC medication (e.g., pain killers). As food products, supplements and OTC products are usually out of scope of healthcare coverage and don't have established efficacy and safety profiles (i.e., frequent off-label use), one can argue to not take these products into consideration when assessing the unmet medical need for OMPs.

#### Criterion—comparative effectiveness and safety (EVIDEM)

Including the criterion “*Comparative* Effectiveness” in MCDA for orphan drugs can be questioned, as per definition there is no approved treatment available in orphan indications, and direct comparisons will rarely happen. Any (indirect) comparator would likely include off-label drugs (often older, generic drugs) and other non-approved treatments, which could limit the validity of such a comparison. It can be argued that the effectiveness or overall “value” of OMPs can be compared with drugs for similar orphan indications or drugs for indications with similar defining properties (e.g., disease type, patient population size). The same goes for comparative safety. Moreover, the efficacy and safety of the drug (and a benefit-risk outcome) have already been established during the marketing authorization application, and in case of an acceptable outcome, an approval (or conditional approval) would be granted. When assessing the efficacy/safety during MCDA one should take the scientific assessments of regulatory agencies into consideration, as clearly contradictory outcomes would be debatable.

#### Criterion—quality of evidence (EVIDEM, trip, kolasa, annemans, garau, paulden)

Generation of robust clinical evidence, endpoint validation and assessing clinical outcomes are inherently problematic in small population groups, which reduces the suitability of “Quality of Evidence” criterion in MCDA assessments for OMPs. Disease severity and progressive disease limits the use of placebo-controlled trials in many rare diseases and data sets will generally be small. Evidence quality by itself would not lead to meaningful differentiation between many (ultra) orphan drugs and could automatically disqualify many orphan drugs if they were to be directly compared to drugs for common diseases with a perceived “adequate” evidence quality. The authors think that a proper approach would be pragmatic and aim for certain flexibility, i.e., to allow for evidential uncertainty when this cannot be reasonably avoided, within pre-defined limits. Regulatory agencies are willing to accept a reduced burden of evidence in certain situations (i.e., for orphan drugs), but often under specific conditions that oblige manufacturers to generate additional evidence on efficacy, safety and practical use (post-approval). Accordingly, payers could approach HTA and reimbursement in case of evidential uncertainty in a similar fashion, i.e., allow early but “conditional reimbursement” for certain promising treatments, with the requirement to generate real-world evidence and perform an updated pharmacoeconomic assessment periodically. An example of a country where conditional reimbursement was used is the Netherlands, but due to the limited number and low quality of applications it has been replaced by a more general subsidy program focused on small and medium-sized enterprises (47).

Garau et al. (45) and Paulden et al. (8) have proposed to include Quality of Evidence as a “multiplier” (or weight) to the effectiveness/outcomes scores, e.g., diminish the effectiveness by a “Quality factor” depending on the certainty of the evidence. While this is an elegant solution, it would create a twice-weighting process for each of the treatment outcomes (e.g., preference weights to the fields and quality of evidence weights to the field scores), and thus complicate the model and interpretation of scores.

#### Criterion—innovativeness and incentives for manufacturers (hughes-wilson, kolasa, annemans, piniazkho)

A major hurdle for orphan drug developers is generating a return on investment, due to the small patient population that will use the final product. However, most HTA models take a societal perspective or a payer-centric view and do not put emphasis on factors that are important for developers of orphan drugs. In contrast, the EU regulatory framework specifically incentivizes orphan drug development by providing a range of regulatory and commercial benefits for OMP manufacturers. To align EU HTA processes with these incentives, MCDA criteria would be needed that represent a similar benefit/valuation for manufacturers of orphan drugs. Several authors have tried to capture “innovation” as a factor, e.g., Gutierrez et al. (51) provided a review of MCDA principles from the manufacturer's perspective and stated that “innovativeness” should be represented in models; Sussex et al. (37) defined the criterion “Treatment innovation: scientific advanced contribution to patient outcome”; Hughes-Wilson et al. (33) included “Level of research” and Annemans et al. (30) considered innovation as one of the key principles to improve consistency of orphan drug pricing and reimbursement assessment.

Kolasa et al. (34) presented a detailed scoring proposal for measuring *manufacturing complexity* via the cost of biotech processes, complexity of drug synthesis based on the number of “chemical transformations” and the necessity to use “separation techniques” for chemical intermediates. To measure “Advancement of technology”, Kolasa et al. (34) made a distinction between Advanced therapy medicinal products (ATMPs), complex drugs, small molecules (with at least one chiral center) and simple chemical entities. While the intent is appreciated, the described methodology to measure innovation has several drawbacks. Firstly, Kolasa et al's. (34) focus lies primarily on rewarding the investment in the manufacturing process, while the bulk of innovation and development costs would typically be related to the clinical trial program. Even for a new indication of a “simple” and well-known molecule, at least a Phase II or Phase III trial program is needed to generate data on safety and effectiveness. Trial costs are relatively high for orphan drugs since trials often need to be run globally over many centers to recruit sufficient numbers of eligible rare disease patients. A lack of clinical (development) guidelines, regulatory precedence, clinical experience, and expertise complicate orphan clinical programs further. Secondly, the authors believe that the chemical properties of the active ingredient do not necessarily represent the level of innovation, treatment success or research and development (R&D) investment. The exact monetary investments and pricing components by manufacturers are difficult to establish and verify externally, especially on the product level: project R&D costs often include broad overhead costs and company-wide write-offs. Alternatively, the authors propose to include the following two, more objectively quantifiable criteria to reward innovation: “Novelty of the active pharmaceutical ingredient” (e.g., defined as first in-class product, second in-class, or existing active ingredient) and “Novelty of the disease indication” (new target population or a subgroup of an existing target population, e.g., a disease subpopulation with certain mutations/biomarkers). One way to measure these would be through the ATC-code of the compound and ICD disease classification. If not considered elsewhere, innovation steps regarding pharmaceutical form improvements could also be considered here, e.g., those that result in increased ease of administration/dosing, but over-rewarding relatively simple R&D investments should be avoided. Together with Population size, these criteria would represent a clear incentive for drug developers.

The type of trial that has been performed (i.e., randomized controlled trial (RCT), open-label trial, use of historical control arms) also represents a type of innovativeness and R&D investment. However, trial design would typically be already taken into account through the Quality of Evidence criterion or weight-factor (where a double-blind RCT represents the golden standard), so double-counting would need to be carefully avoided if this were to be considered.

### Economic consequences of intervention

#### Separating cost-components from the value-components of the intervention

A possible methodological flaw in several MCDA models is the incorporation of both costs and value (benefits) into one aggregated MCDA end-score, i.e., one additive score containing disease characteristics, treatment outcomes and cost-related criteria. Using such an aggregate score creates difficulties in assessing and interpreting the final MCDA outcome and complicates comparison of technologies. Since economic consequences are highly dependent on the local healthcare system and economic factors (e.g., GDP/healthcare budget/reimbursement structure/availability of care), separating criteria that define the “Value of the intervention” from those defining “Economic consequences” (e.g., costs) would allow for generating more objective, meaningful and understandable MCDA scores. Having a distinct “Value-Score” and “Cost-Score” of the treatment could simplify models, ease interpretation and create the flexibility to easily transfer outcomes to the national HTA level (transferability). For example, a treatment's value-score (for use in HTA) could be assessed centrally by the EMA or any other appointed scientific/regulatory and be transferred to the national HTA body for use in MCDA or other methods. The selection and definition of cost-related factors for HTA and the actual cost-calculation is performed nationally, where the cost- and value-scores can be weighed for decision-making on local healthcare coverage. Such an approach would also be aligned with the EU proposal for a new HTA Regulation (16) which describes a centralized “joint clinical assessment” (JCA). A JCA at the European level enables sharing of HTA workload between member states, similar to regulatory assessments in the EMA Centralized Procedure. Especially for orphan drugs, a centralized clinical (value) assessment by highly skilled medical and OMP HTA experts could improve the quality and speed of national health technology assessments. Objectively established value-scores could also be used for relatively quick and easy value-comparison of treatments, without bringing in cost-factors. Paulden et al. (8) used a similar approach in their model design.

#### Type of economic analysis

How to balance value vs. costs in healthcare budgets is one of the key considerations made by several researchers, especially whether the economic assessment should be based on opportunity-cost analysis or more traditional cost-effectiveness analysis. In situations where budgets are finite, as in healthcare systems, opportunity-cost analysis allows for the evaluation of resources that cannot be spent due to expenditure elsewhere, which stimulates the “replacement” of older interventions by newer ones with a lower opportunity cost. The opportunity cost approach plays a central role in Paulden et al's. (8) decision framework, in which the “Net Value” is the total treatment valuation minus opportunity cost (vs. relevant comparators). The Lombardian VTS framework also explicitly promotes a mandatory “delisting” of older, obsolete technologies via an opportunity-cost approach (28).

### Other methodology considerations

Multiple mathematical and statistical methods can be used for scoring, weighting, analyzing and comparing alternatives in MCDA models. The theoretical basis for the models is rarely discussed in most of the reviewed studies. An ISPOR task force has issued 2 publications on best practices in MCDA to guide model developers in 2016 (19, 61). However, since most MCDA models are older than 2016 they are not following these guidelines yet, e.g., regarding non-overlapping criteria. Broekhuizen et al. (62) identified the 5 most common approaches for dealing with uncertainty in MCDA models (such as fuzzy set theory), but these are not discussed by any of the model creators (62). A study on the practical applicability of MCDA in Canada found that the quantification of evidence and interpretation of the aggregate MCDA score was “challenging” and that comparing/ranking interventions would require a better grasp of the underlying methodology (63). Gandjour (60) commented on the EVIDEM framework and expressed that the model in its current state is an “intermediate” solution and several improvements should be made, including an independent ethical justification and stronger theoretical foundation, especially for the use of individual preferences (60).

A recent EU survey (63) shows that a wide range of factors are taken into account by HTA agencies, e.g., 63% include social aspects such as “burden on care-givers.” A certain flexibility in data quality requirements is visible, as 80% of agencies are reported to accept prospective, non-randomized studies or other kinds of observational studies, traditionally viewed as being of lower quality. Similarly, 90% can accept surrogate endpoints for effectiveness or safety and around 80% acknowledge PROs, Health-Related Quality of Life measures and indirect comparisons if head-to-head comparator trials are lacking. Furthermore, 71% of agencies accept composite endpoints and network meta-analysis while 96% acknowledge subgroup analyses (63). These findings show that many HTA agencies assess non-traditional factors, which could serve as a basis for introducing MCDA into healthcare HTA.

## Conclusions

Over the last 10 years a range of MCDA models for HTA have been created, each with a slightly different approach, focus and complexity, including several models that specifically target rare diseases and orphan drugs. These models have slowly progressed based on pilots, stakeholder input, sharing experiences and scientific publications. However, full consensus on model structure, criteria selection, and weighting is still lacking. As shown in this and other publications, a more fundamental discussion on the methodological and mathematical design aspects of MCDA models is ongoing and needed. Initiatives such as the EVIDEM framework and the MoCA project help to create momentum and standardization, but have not led to any significant EU-wide implementation. This shows that apparently hurdles still need to be overcome and model improvements are necessary. The planned EU HTA regulation could have some positive spill-over effects to stimulate the development and harmonization of MCDA in HTA. Given the differences in national healthcare and reimbursement systems, as well as local variations in economy and rare disease policies, HTA models that are flexible and adaptable would be required. MCDA can capture factors beyond standard cost-effectiveness analysis and offer a range of possibilities that would be suitable for rare disease HTA.

The authors have attempted to highlight some of the problems that exist with the design of current MCDA models, with a review of commonly used model criteria and how to generate more clear and meaningful results with them. As it stands, models are often unclear and results difficult to interpret, which warrants a simplification of current designs with fewer and well-defined domains and with less overlap between criteria. A strict separation of value from costs in MCDA models would be increase the flexibility, clarity of the model, and transferability of the results, which could aid implementation. Further research, model improvement and validation, practical application and multi-stakeholder discussion are necessary to bring about consensus and to fulfill the potential that MCDA promises in healthcare HTA.

## Author contributions

AB-K contributed to conceptualization, research, methodology, writing—original draft preparation, review, and editing. MC contributed to methodology, supervision, writing—review, and editing. CK contributed to writing—review, research, and editing.

### Conflict of interest statement

The authors declare that the research was conducted in the absence of any commercial or financial relationships that could be construed as a potential conflict of interest.

## Footnotes

^1^https://www.geneticalliance.org.uk/media/2502/hidden-costs-full-report_21916-v2-1.pdf (Accessed March 18, 2018).

^2^https://www.nice.org.uk/Media/Default/About/what-we-do/NICE-guidance/NICE-highly-specialised-technologies-guidance/HST-interim-methods-process-guide-may-17.pdf (Accessed August 29, 2018).

^3^https://www.nice.org.uk/about/what-we-do/our-programmes/nice-guidance/nice-highly-specialised-technologies-guidance (Accessed 29, 2018).

^4^http://skc-beratung.de/wp-content/uploads/2017/03/White_Paper_SKC.pdf (Accessed 29, 2018).

^5^https://www.evidem.org/ (Accessed March 18, 2018).

^6^https://www.eurordis.org/content/moca (Accessed March 18, 2018).

## References

[1] Drummond MF Challenges in the economic evaluation of orphan drugs. Eurohealth (2008) 14:16–17. Available online at: http://www.lse.ac.uk/lse-health/assets/documents/eurohealth/issues/eurohealth-v14n2.pdf?from_serp=1

[2] Simoens S Health technologies for rare diseases: does conventional HTA still apply? Expert Rev Pharmacoecon Outcomes Res. (2014) 14:315–7. 10.1586/14737167.2014.906903 24702042

[3] Walker A Challenges in using MCDA for reimbursement decisions on new medicines? Value Health (2016) 19:123–4. 10.1016/j.jval.2016.02.001 27021744

[4] Vorobiev P Holownia M Krasnova L Multi-criteria decision analysis (MCDA) and its alternatives in health technology assessment, JHPOR (2015) 1:34–43. 10.7365/JHPOR.2015.1.4

[5] U.S. Food and Drug Administration, Pariser A Rare Disease and Clinical Trials (04.11.2014). Available online at: https://www.fda.gov/downloads/Drugs/NewsEvents/UCM440797.pdf (Accessed March 18, 2018).

[6] Hilgers RD König F Molenberghs G Senn S Design and analysis of clinical trials for small rare disease populations. J Rare Dis Res Treat. (2016) 1:53–60. 10.29245/2572-9411/2016/3.1054

[7] Schuller Y Hollak CEM Biegstraaten M. The quality of economic evaluations of ultra-orphan drugs in Europe – a systematic review. Orphanet J Rare Dis. (2015) 10:92. 10.1186/s13023-015-0305-y 26223689 PMC4520069

[8] Paulden M Stafinski T Menon D McCabe C. Value-based reimbursement decisions for orphan drugs: a scoping review and decision framework. Pharmacoeconomics (2014) 33:255–69. 10.1007/s40273-014-0235-x 25412735 PMC4342524

[9] Kodra Y Cavazza M Schieppati A De Santis M Armeni P Arcieri R. The social burden and quality of life of patients with haemophilia in Italy. Blood Transfus. (2014) 12(Suppl. 3):s567–75. 10.2450/2014.0042-14s 24922297 PMC4044804

[10] Angelis A Kanavos P López-Bastida J Linertová R Juan Oliva-Moreno Pedro Serrano-Aguilar P and BURQOL-RD Research Network. Social/economic costs and health-related quality of life in patients with epidermolysis bullosa in Europe. Eur J Health Econ. (2016) 17(Suppl. 1):31–42. 10.1007/s10198-016-0780-7 PMC4869727 27107597

[11] Angelis A Tordrup D Kanavos P. Socio-economic burden of rare diseases. a systematic review of cost of illness evidence. Health Policy (2015) 119:964–79. 10.1016/j.healthpol.2014.12.016 25661982

[12] Baran A Czech M Hermanowski T Skoczynska K Direct and indirect costs of Pompe disease. Farmacja Współczesna (2014) 7:149–55. Available online at: https://www.akademiamedycyny.pl/wp-content/uploads/2016/05/201404_Farmacja_001.pdf

[13] Blankart CR Koch T Linder R Verheyen F Schreyögg J Stargardt T. Cost of illness and economic burden of chronic lymphocytic leukemia. Orphanet J Rare Dis. (2013) 8:32. 10.1186/1750-1172-8-32 23425552 PMC3598307

[14] Gammie T Lu CY Babar ZU. Access to orphan drugs. a comprehensive review of legislations, regulations and policies in 35 countries. PLoS ONE 10:e0140002. 10.1371/journal.pone.0140002 26451948 PMC4599885

[15] Libura M Władysiuk M Małowicka M Grabowska E Gałazka-Sobotka M Gryglewicz J Rare Disease in Poland, Current Status and Perspectives; Choroby rzadkie w Polsce, Stan Obecny i Perspektywy. Uczelnia Łazarskiego (2016)

[16] Belgin G Macarthur D Access to Orphan Drugs in Turkey. Jan 04, (2016)

[17] Kiliç P Koçkaya G Yemşen Ö Tan C Handan Öztunca F Aksungur P Orphan drug regulation in Turkey. JPHSR (2013) 4:151–3. 10.1111/jphs.12018

[18] Weinstein N Martin M Campbell R Orphan drugs in the Uk, do they meet the nice highly specialised technology threshold? Value Health (2017) 20:A660 10.1016/j.jval.2017.08.1581

[19] Thokala P Devlin N Marsh K Baltussen R Boysen M Kalo Z et al Multiple criteria decision analysis for health care decision making—an introduction:report 1 of the ISPOR MCDA emerging good practices task force. Value Health (2016) 19:1–13. 10.1016/j.jval.2015.12.003 26797229

[20] Keeney RL Raiffa H Decisions with Multiple Objectives: Preferences and Value Trade-Offs. Cambridge: Cambridge University Press (1993).

[21] Carver SJ Integrating multi-criteria evaluation with geographical information systems. Int J Geogr Inf Syst. (1991) 5:321–39. 10.1080/02693799108927858

[22] Hallerbach W Spronk J The relevance of MCDM for financial decisions. J Multi-Criteria Decis Anal. (2003) 11:187–95. 10.1002/mcda.328

[23] Linkov I Moberg E Multi-Criteria Decision Analysis: Environmental Applications and Case Studies. Boca Raton, FL: CRC Press, Taylor & Francis Group (2011).

[24] Wagner M Khoury H Levitt RJ Erickson LJ Rindress D Evidence and value: impact on decision Making – the EVIDEM framework and potential applications. BMC Health Serv Res. (2008) 8:270 10.1186/1472-6963-8-270 19102752 PMC2673218

[25] Wagner M Khoury H Willet J Rindress D Goetghebeur M. Can the EVIDEM Framework tackle issues raised by evaluating treatments for rare diseases: analysis of issues and policies, and context-specific adaptation. PharmacoEconomics (2016) 34:285–301. 10.1007/s40273-015-0340-5 26547306 PMC4766242

[26] Hughes-Wilson W MoCA Concept and Pilot Project, Feedback from the process around the first pilot project, ECRD Berlin (10.05.2014). Available online at: http://download2.eurordis.org.s3.amazonaws.com/moca/presentations/PRES-2014-05%20MoCA%20Concept%20and%20Pilot%20Project%20(Hughes-Wilson).pdf (Accessed March 18, 2018).

[27] Endrei D Molics B Ágoston I. Multicriteria decision analysis in the reimbursement of new medical technologies: real-world experiences from Hungary. Value Health (2014) 17:487–9. 10.1016/j.jval.2014.01.011 24969012

[28] Radaelli G Lettieri E Masella C Merlino L Strada A Tringali M. Implementation of EUnetHTA core Model® in Lombardia: the VTS framework. Int J Technol Assess Health Care (2014) 30:105–12. 10.1017/S0266462313000639 24451150

[29] Marsh K Wagner M Thokala P Baltussen R Multi-Criteria Decision Analysis to Support Healthcare. Cham: Springer (2017). 10.1007/978-3-319-47540-0

[30] Annemans L Aymé S Le Cam Y Facey K Gunther P Nicod E. Recommendations from the European working group for value assessment and funding processes in rare diseases (ORPH-VAL) Orphanet J Rare Dis. (2017) 12:50. 10.1186/s13023-017-0601-9 28283046 PMC5345269

[31] Palaska C Hutchings A. Value assessment and pricing frameworks for rare disease treatments: new approaches from the literature. PSY113. Value Health (2015) 18:A678. 10.1016/j.jval.2015.09.201326533803

[32] Friedmann C Levy P Hensel P Hiligsmann M. Using multi-criteria decision analysis to appraise orphan drugs: a systematic review. Expert Rev Pharmacoeconom Outcomes Res. (2018) 18:135–46. 10.1080/14737167.2018.1414603 29210308

[33] Hughes-Wilson W Palma A Schuurman A Simoens S. Paying for the Orphan Drug System: break or bend? Is it time for a new evaluation system for payers in Europe to take account of new rare disease treatments? Orphanet J Rare Dis. (2012) 7:74. 10.1186/1750-1172-7-74 23013790 PMC3582462

[34] Kolasa K Zwolinski KM Kalo Z Hermanowski T Potential impact of the implementation of multiple-criteria decision analysis (MCDA) on the Polish pricing and reimbursement process of orphan drugs *Orphanet* J Rare Dis. (2016) 11:23 10.1186/s13023-016-0388-0 PMC4787054 26965710

[35] Iskrov G Miteva-Katrandzhieva T Stefanov R. Multi-criteria decision analysis for assessment and appraisal of Orphan Drugs. Front. Public Health (2016) 4:214. 10.3389/fpubh.2016.00214 27747207 PMC5042964

[36] Trip AM Tsiachristas A Koenders JM Kanters TA. Multi-criteria decision analysis for reimbursing orphan drugs: a Dutch demonstration study using the analytic hierarchy process method. PSY 114, Value Health (2014) 17:A541–2. 10.1016/j.jval.2014.08.1744 27201744

[37] Sussex J Rollet P Garau M Schmitt C Kent A Hutchings A BRIEF REPORTS A pilot study of multicriteria decision analysis for valuing orphan medicines. Value Health (2013) 16:1163–9. 10.1016/j.jval.2013.10.002 24326170

[38] Fedyaeva VK Omelyanovsky VV Rebrova O Khan N Petrovskaya EV. Mcda approach to ranking rare diseases in Russia: preliminary, PSY99. Value Health (2014) 17:A539. 10.1016/j.jval.2014.08.1729 27201727

[39] Fedyaeva VK Omelyanovskiy VV Rebrova OY Marsh K PSY121 - Comparison of methods to assess the relative importance of criteria in multi-criteria decision analysis: an evaluation of orphan drugs in Russia. Value Health (2016) 19:A596–6. 10.1016/j.jval.2016.09.1438

[40] Piniazhko O Zalis'ka O Brezden O Methodological issues in MCDA for training needed: eliciting stakeholders' value preferences in preferences in Ukraine. Value Health (2017) 20:A45.

[41] Piniazhko O Nemeth B Practical issues of determining weights for criteria to be used in an MCDA framework-based on a case-study Value Health (2017) 20:A51–2.

[42] Schey C Irwin J Teneishvili M Krabbe PFM Connolly M. Assessing the relationship between individual attributes identified in review of Multi-Criteria Decision Analysis (MCDA) of rare diseases and annual treatment costs in rare endocrine disorders, PRM108. Value Health (2014) 17:A323–686 27201857 10.1016/j.jval.2014.08.1860

[43] Wagner M Khoury H Bennetts L Berto P Ehreth J Badia X. Appraising the holistic value of lenvatinib for radio-iodine refractory differentiated thyroid cancer: a multi-country study applying pragmatic. BMC Cancer (2017) 17:272. 10.1186/s12885-017-3258-9 28412971 PMC5393009

[44] Gilabert-Perramon A Torrent-Farnell J Catalan A Prat A Fontanet M Puig-Peiró R. Drug evaluation and decision making in Catalonia: development and validation of a methodological framework based on multi-criteria decision analysis (MCDA) for orphan drugs. Int J Technol Assess Health Care (2017) 33:111–20. 10.1017/S0266462317000149 28434413

[45] Garau M Marsden G Devlin N Amedeo Mazzanti N Profico A Applying a multi-criteria decision analysis (MCDA) approach to elicit stakeholders' preferences in Italy. The case of obinutuzumab for rituximab-refractory indolent Non-Hodgkin Lymphoma (iNHL). Office of Health Economics Res. Research Paper 16/08, December (2016).

[46] Schey C Krabbe PFM Postma MJ Connolly MP. Multi-criteria decision analysis (MCDA):testing a proposed MCDA framework for orphan drugs. Orphanet J Rare Dis. (2017) 12:10. 10.1186/s13023-016-0555-3 28095876 PMC5240262

[47] Tony M Goetghebeur MM Khoury H Wagner M Deal CL Battista R A common road map for rational clinical and policy decisionmaking: application of the mcda-based evidem framework to growth hormone use in patients with prader-willi syndrome. Value Health (2011) 14:A328 10.1016/j.jval.2011.08.525

[48] Jimenez A Ais A Acuña L González ME Paco N Gil A Determining the value of selexipag for the treatment of pulmonary arterial hypertension (PAH) in Spain by multi-criteria decision analysis (MCDA). Value Health (2017) 20:A570 10.1016/j.jval.2017.08.971 PMC6288887 30526673

[49] Krysanova V Krysanov I Ermakova V The multicriteria decision analysis of using tetrabenazine for patients with hungtington's disease in Russia. Value Health (2017) 20:A565 10.1016/j.jval.2017.08.945

[50] Badia X Pontes C Fontanet M Obach M Vallano A Torrent J PHP129 - development of an specific evaluation framework for orphandrugs based on Multi-Criteria Decision Analysis (MCDA) for health care decision making in Catalonia. Value Health (2017) 20:A674–4. 10.1016/j.jval.2017.08.1662

[51] Gutierrez L Patris J Hutchings A Cowell W. Principles for consistent value assessment and sustainable funding of orphan drugs in Europe. Orphanet J Rare Dis. (2015) 10:53. 10.1186/s13023-015-0269-y 25935555 PMC4433088

[52] Schlander M Garattini S Holm S Kolominsky-Rabas P Marshall DA Nord E Interventions for Ultra-Rare Disorders (URDs) and the logic of cost effectiveness. Value Health (2015) 18:A6 10.1016/j.jval.2015.03.043 27200532

[53] Zhang A Weisse S Chen X Health Technology Assessment (HTA) for orphan drugs in cost-effectiveness (CE) markets: current development and future trends. Value Health (2016) 19:A601 10.1016/j.jval.2016.09.1464

[54] Nemeth B, Piniazhko O. Mcda application in central and eastern Europe: selection of the most important criteria based on examples. Value Health (2016) 19:A471 10.1016/j.jval.2016.09.723

[55] Korchagina D Millier A Toumi M Falissard B Elements of orphan drugs value. Value Health (2016) 19:A600–1. 10.1016/j.jval.2016.09.1463

[56] Hutchings A Ethgen O Schmitt C Rollet P Defining elements of value for rare disease treatments. Value Health (2012) 15:A31.

[57] Goetghebeur MM Wagner M Samaha D O'Neil W Badgley D Castro-Jaramillo H. Exploring values of health technology assessment agencies using reflective multicriteria and rare disease case. Int J Technol Assess Health Care (2017) 33:504–20. 10.1017/S0266462317000915 29019295

[58] Tony M Wagner M Khoury H Rindress D Papastavros T Oh P. Bridging health technology assessment (HTA) with multicriteria decision analyses (MCDA): field testing of the EVIDEM framework for coverage decisions by a public payer in Canada. BMC Health Serv Res. (2011) 11:329. 10.1186/1472-6963-11-329 22129247 PMC3248909

[59] Angelis A Kanavos P. Multiple Criteria Decision Analysis (MCDA) for evaluating new medicines in health technology assessment and beyond: the advance value framework. Soc Sci Med. (2017) 188:137–56. 10.1016/j.socscimed.2017.06.024 28772164

[60] Gandjour A. Comment on: “Can the EVIDEM framework tackle issues raised by evaluating treatments for rare diseases: analysis of issues and policies, and context-specific adaptation”. Pharmacoeconomics (2017) 35:603–4. 10.1007/s40273-017-0493-5 28324438

[61] Marsh K IJzerman M Thokala P Baltussen R Boysen M Kaló Z. Multiple criteria decision analysis for health care DECISION Making—emerging good practices: report 2 of the ISPOR MCDA emerging good practices task force. Value Health (2016) 19:125–37. 10.1016/j.jval.2015.12.016 27021745

[62] Broekhuizen H Groothuis-Oudshoorn CG van Til JA Hummel JM IJzerman MJ. A review and classification of approaches for dealing with uncertainty in multi-criteria decision analysis for healthcare decisions. Pharmacoeconomics (2015) 33:445–5. 10.1007/s40273-014-0251-x 25630758 PMC4544539

[63] European Commission Finn Børlum Kristensen Mapping of HTA methodologies in EU and Norway (2017). Available online at: https://ec.europa.eu/health/sites/health/files/technology_assessment/docs/2018_mapping_methodologies_en.pdf (Accessed March 18, 2018).

